# Editorial: With obesity becoming the new normal, what should we do? ‐Volume II

**DOI:** 10.3389/fendo.2022.1119910

**Published:** 2022-12-28

**Authors:** Konstantinos Tziomalos, Tuomas O. Kilpeläinen, Katherine Samaras

**Affiliations:** ^1^ First Propedeutic Department of Internal Medicine, Medical School, Aristotle University of Thessaloniki, AHEPA Hospital, Thessaloniki, Greece; ^2^ Novo Nordisk Foundation Center for Basic Metabolic Research, Faculty of Health and Medical Sciences, University of Copenhagen, Copenhagen, Denmark; ^3^ Department of Endocrinology, St Vincent’s Hospital, Darlinghurst, NSW, Australia; ^4^ Clinical Obesity, Nutrition and Adipose Biology Lab, Clinical Science Pillar, Garvan Institute of Medical Research, Darlinghurst, NSW, Australia; ^5^ St Vincent’s Clinical School, Faculty of Medicine, University of New South Wales, Darlinghurst, NSW, Australia

**Keywords:** obesity, cardiometabolic health, diet, cardiovascular disease, overweight

The prevalence of obesity is increasing worldwide and in many regions, normal weight people have become the minority (1). This pandemic is already translating into increased incidence of cardiometabolic disease, cancer and other obesity-related sequelae (2). Notably, hypertension, dyslipidemia and diabetes mellitus account only for half of the increased cardiovascular risk in obese patients suggesting that weight loss is essential for addressing the residual risk (3). In the present special issue, a series of papers provide the reader with novel insights regarding obesity and its related comorbidities.

Nonalcoholic fatty liver disease (NAFLD) is the commonest chronic liver disease in high-income countries and is closely related to obesity (4). Diet and exercise is the cornerstone of management of NAFLD but only a minority of patients achieves substantial and sustained weight loss with lifestyle changes (5). Therefore, weight loss agents are frequently considered in these patients. Tsankof et al. summarize the limited evidence regarding the safety and efficacy of pharmacotherapy for obesity in patients with NAFLD. It appears that glucagon-like peptide-1 receptor agonists represent the treatment of choice for this population given their safety and efficacy. However, their effects on liver fibrosis are unclear, and it has not yet been evaluated whether these agents improve the liver-related and cardiovascular morbidity of patients with NAFLD.

Hyperuricemia is another common comorbidity in patients with obesity (6, 7). In this context, Cheang et al. report that 5.1, 15.2, 16.9 and 32.5% of Chinese patients with body mass index 25.0-29.9, 30.0-34.9, 35.0-39.9 and ≥ 40.0 kg/m^2^ have elevated serum uric acid levels. Given the high prevalence of hyperuricemia in obese patients and its association with the risk of gout, it appears prudent to measure uric acid levels in this population, particularly in patients with severe obesity.

Bariatric surgery is a treatment option for patients with severe obesity and obesity-related comorbidities (8). Roux-en-Y gastric bypass surgery is one of the most frequently performed methods of bariatric surgery (8). However, the optimal size of gastric pouch that is created during this surgery remains unclear (8). Gao et al. performed a retrospective study to address this question and show that a small gastric pouch results in greater weight loss, better glycemic control and a lower rate of marginal ulcers. Accordingly, creating a small gastric pouch during Roux-en-Y gastric bypass surgery appears to be more effective and safer than creating a larger pouch. However, larger prospective studies are needed to validate these findings.

The therapeutic potential of microbiome manipulation to mitigate the impact of obesity is explored in this Research Topic with a review from Lee et al. This excellent review presents the reader with the latest research understanding that builds on knowledge on microbiome phenotypic/genotypic composition to functional aspects of metabolites, including small chain fatty acids, bile acids and tryptophan metabolites, and how these may interact with central and peripheral pathways that regulate metabolism. A particular aspect of this review that will appeal to readers is the synthesis of data examining how gut metabolites are involved in the pathophysiology of obesity and its associated complications.

How do parents create a healthy living environment for their children? How do they address this challenge in a commercial environment that promotes overconsumption of energy dense foods and beverages and encourages sedentariness? The Confident Body, Confident Child, a universal program aimed at preventing childhood obesity and disturbed eating and body image in children, aims to create parent lifestyle champions for healthy, confident children. The paper by Fiskum et al. reports qualitative research from parent and professional focus groups, with the aim of adapting the program into a Norwegian-specific translation. The investigators report high levels of parental stress related to an “all-or-nothing” dichotomy of healthy eating versus not. Further, parents reported shame when they perceived their own failures and, importantly, disengagement. These findings stimulate us to consider health messaging and the challenges not only of parents, but all of us trapped in an environment of energy dense foods and beverages, where a binary of “good” versus “bad” is applied and healthy eating is privileged to a virtue. Further research is awaited on how to best enable parents in negotiating the energy dense food environment we are trapped in, and to create environments for healthy confident children who recapitulate the model in future generations.

Sodium-glucose cotransporter-2 inhibitors (SGLT2i) and glucagon-like peptide-1 receptor agonists (GLP-1RA) are recently introduced glucose-lowering drugs that also induce weight loss. The weight loss occurring duringSGLT2i treatment has been attributed to urinary glucose excretion. However, the weight loss is smaller than would be expected from the amount of glucose excreted into urine. It has therefore been hypothesized that SGLT2i treatment may increase energy intake. Using functional MRI data from a randomized clinical trial, Ruiten et al. show that theSGLT2i dapagliflozin lowers brain response to low-calorie food cues and increases response to high-calorie cues. In contrast, treatment with the GLP-1RA exenatide increases brain response to low-calorie food cues and decreases response to high-calorie cues. Ruiten et al. also find that combining dapagliflozin with exenatide normalizes brain responses to the low and high-calorie food cues. The authors discuss that a combined treatment may help optimize weight loss.

Overweight and obesity are associated with a high prevalence of impaired glucose tolerance, which can be treated effectively with lifestyle modifications and glucose-lowering medications. Acupuncture has been proposed as an alternative treatment for impaired glucose tolerance. However, the beneficial effect of acupuncture has not yet been tested in a clinical trial. Yan et al. describes the study design of a randomized clinical trial on the effect of acupuncture on blood two-hour plasma glucose levels in 196 individuals randomly assigned to an acupuncture or a sham-acupuncture group. Eachgroup will receive 30 acupuncture sessions over 12 consecutive weeks.The primary outcome will be the change in 2-hour glucose level over the intervention. The trial will help resolve whether acupuncture can bean effective and safe treatment for impaired glucose tolerance in persons with overweight or obesity.

Finally, the impact of immediate and abrupt lifestyle change imposed by lockdown measures to reduce the spread of coronavirus on weight gain was modelled by Murphy et al. Using data from the Canadian Community Health Study collected in the years immediately before the pandemic, simulated models estimated weight changes that could be attributed to the impact of the pandemic and from there, additional incident cancers in the ensuing 10, 15 and 20 years. At 20 years, more than 14,000 additional incident cancers were attributed to pandemic weight gain, disproportionately affecting women and breast cancer rates and people aged greater than 60 years. This study serves as an interesting model to be applied based on recent epidemiological data meticulously collected. Mathematical models of these kinds are useful to health policy makers in considering future health screening and health resource allocation, particularly for cancer. The outputs from this study are prescient and demand urgent strategies to reverse the weight gain made by many in the modern era of the obesity pandemic.

In conclusion, the increasing prevalence of obesity poses major challenges. The management of obesity-related cardiometabolic risk factors is essential but not sufficient for negating the cardiovascular risk of obese patients. Safe and effective pharmacological agents are needed to achieve and sustain weight loss. In addition, novel, population-based strategies are needed to prevent weight gain and maintain a healthy weight, particularly in children and adolescents.

## Author contributions

KT drafted the editorial. TK and KS critically revised the draft. All authors approved the final draft.

## References

[1] NCD Risk Factor Collaboration (NCD-RisC) . Worldwide trends in body-massindex, underweight, overweight, and obesity from 1975 to 2016: a pooled analysis of 2416 population-based measurement studies in 128·9 million children, adolescents, and adults. Lancet (2017) 390:2627–42. doi: 10.1016/S0140-6736(17)32129-3 PMC5735219 29029897

[2] Wang YC McPherson K Marsh T Gortmaker SL Brown M . Health and economic burden of the projected obesity trends in the USA and the UK. Lancet (2011) 378:815–25. doi: 10.1016/S0140-6736(11)60814-3 21872750

[3] Global Burden of Metabolic Risk Factors for Chronic Diseases Collaboration (BMI Mediated Effects) Lu Y Hajifathalian K Ezzati M Woodward M Rimm EB . Metabolic mediators of the effects of body-mass index, overweight, and obesity on coronary heart disease and stroke: a pooled analysis of 97 prospective cohorts with 1·8 million participants. Lancet (2014) 383:970–83. doi: 10.1016/S0140-6736(13)61836-X PMC3959199 24269108

[4] Sarwar R Pierce N Koppe S . Obesity and nonalcoholic fatty liver disease: Current perspectives. Diabetes Metab Syndr Obes (2018) 11:533–42. doi: 10.2147/DMSO.S146339 PMC6163009 30288073

[5] Vilar-Gomez E Martinez-Perez Y Calzadilla-Bertot L Torres-Gonzalez A Gra-Oramas B Gonzalez-Fabian L . Weight loss through lifestyle modification significantly reduces features of nonalcoholic steatohepatitis. Gastroenterology (2015) 149:367–378.e5;quiz e14-5. doi: 10.1053/j.gastro.2015.04.005 25865049

[6] Rospleszcz S Dermyshi D Müller-Peltzer K Strauch K Bamberg F Peters A . Association of serum uric acid with visceral, subcutaneous and hepatic fat quantified by magnetic resonance imaging. Sci Rep (2020) 10:442. doi: 10.1038/s41598-020-57459-z 31949261 PMC6965096

[7] Ogura T Matsuura K Matsumoto Y Mimura Y Kishida M Otsuka F . Recent trends of hyperuricemia and obesity in Japanese male adolescents, 1991 through 2002. Metabolism (2004) 53:448–53. doi: 10.1016/j.metabol.2003.11.017 15045690

[8] Buchwald H Buchwald JN . Metabolic (Bariatric and nonbariatric) surgery for type 2 diabetes: A personal perspective review. Diabetes Care (2019) 42:331–40. doi: 10.2337/dc17-2654 30665965

